# Supplementary material for: Halotolerance of Phytoplankton and Invasion Success of Nostocalean Cyanobacteria Under Freshwater Salinization
Source: Microorganisms. 2025 Jun 13;13(6):1378. doi: 10.3390/microorganisms13061378 (PMC12195237; doi:10.3390/microorganisms13061378)
Supplement: Supplementary file 1 [file microorganisms-13-01378-s001.zip › microorganisms-3647238-supplementary.pdf]

Table S1. The biomass (mg/L) and relative biomass (%) of identified taxa of phytoplankton in the control group in water body 1.

| Phylum and subphylum | Day 1 | | Last day | | | | | | | |
|---|---|---|---|---|---|---|---|---|---|---|
| | | | 0 g/L NaCl | | 0.2 g/L NaCl | | 1 g/L NaCl | | 5 g/L NaCl | |
| | Biomass, mg/L | Biomass, % | Biomass, mg/L | Biomass, % | Biomass, mg/L | Biomass, % | Biomass, mg/L | Biomass, % | Biomass, mg/L | Biomass, % |
| **Cyanobacteria** | | | | | | | | | | |
| *Chroococcus* | 0.005 | 0.192 | 0.000±0.00 | 0.00±0.00 | 0.045±0.03 | 1.799±1.12 | 0.028±0.00 | 2.197±0.64 | 0.025±0.01 | 4.816±2.05 |
| *Planktolyngbya* | 0.000 | 0.000 | 0.010±0.00 | 0.752±0.55 | 0.011±0.01 | 0.470±0.35 | 0.000±0.00 | 0.011±0.02 | 0.001±0.00 | 0.138±0.24 |
| *Romeria* | 0.000 | 0.000 | 0.004±0.00 | 0.270±0.07 | 0.009±0.01 | 0.310±0.22 | 0.001±0.00 | 0.069±0.11 | 0.000±0.00 | 0.000±0.00 |
| **Heterokontophyta subphylum Ochrophytina** | | | | | | | | | | |
| *Bitrichia* | 0.002 | 0.084 | 0.000±0.00 | 0.018±0.01 | 0.000±0.00 | 0.003±0.01 | 0.000±0.00 | 0.000±0.00 | 0.000±0.00 | 0.000±0.00 |
| *Dinobryon* | 0.016 | 0.668 | 0.000±0.00 | 0.000±0.00 | 0.000±0.00 | 0.000±0.00 | 0.000±0.00 | 0.000±0.00 | 0.000±0.00 | 0.000±0.00 |
| Unidentified | 0.343 | 14.492 | 0.335±0.08 | 21.729±4.10 | 0.810±0.27 | 31.267±3.93 | 0.000±0.00 | 0.000±0.00 | 0.000±0.00 | 0.000±0.00 |
| **Heterokontophyta subphylum Bacillariophytina** | | | | | | | | | | |
| *Cyclotella* | 0.480 | 20.309 | 0.807±0.37 | 48.954±6.20 | 1.126±0.17 | 44.624±3,649 | 1.010±0.19 | 77.97±0.48 | 0.248±0.12 | 46.689±1.02 |
| *Nitzschia* | 0.001 | 0.040 | 0.042±0.01 | 2.924±1.63 | 0.087±0.02 | 3.397±0.72 | 0.025±0.01 | 1.903±0.23 | 0.028±0.04 | 4.156±4.59 |
| *Synedra* | 0.011 | 0.465 | 0.129±0.06 | 7.963±1.98 | 0.139±0.08 | 5.530±3.22 | 0.010±0.01 | 0.704±0.33 | 0.008±0.01 | 1.004±1.74 |
| **Heterokontophyta subphylum Coscinodiscophytina** | | | | | | | | | | |
| *Aulacoseira* | 0.039 | 1.657 | 0.022±0.01 | 1.339±0.51 | 0.042±0.02 | 1.588±0.81 | 0.003±0.01 | 0.231±0.40 | 0.011±0.01 | 2.606±2.28 |
| **Chlorophyta subphylum Chlorophytina** | | | | | | | | | | |
| *Monoraphidium* | 0.007 | 0.308 | 0.009±0.00 | 0.658±0.34 | 0.016±0.01 | 0.609±0.09 | 0.017±0.01 | 1.339±0.26 | 0.023±0.01 | 4.208±0.90 |
| *Nephrochlamys* | 0.104 | 4.411 | 0.041±0.01 | 2.618±0.32 | 0.058±0.03 | 2.267±0.83 | 0.042±0.01 | 3.365±0.90 | 0.001±0.00 | 0.270±0.26 |
| *Oocystis* | 0.012 | 0.501 | 0.000±0.00 | 0.000±0.00 | 0.000±0.00 | 0.000±0.00 | 0.002±0.00 | 0.152±0.26 | 0.000±0.00 | 0.000±0.00 |
| *Pediastrum* | 0.000 | 0.000 | 0.008±0.01 | 0.402±0.55 | 0.002±0.00 | 0.051±0.09 | 0.003±0.01 | 0.219±0.38 | 0.000±0.00 | 0.000±0.00 |
| *Scenedesmus* | 0.066 | 2.780 | 0.182±0.09 | 10.869±1.70 | 0.175±0.04 | 6.875±0.52 | 0.039±0.01 | 2.939±0.52 | 0.048±0.02 | 9.590±2.02 |
| *Tetraedron* | 0.032 | 1.355 | 0.007±0.01 | 0.411±0.19 | 0.011±0.00 | 0.455±0.22 | 0.006±0.01 | 0.420±0.40 | 0.010±0.01 | 3.00±3.52 |
| *Tetrastrum* | 0.009 | 0.401 | 0.002±0.00 | 0.131±0.12 | 0.001±0.00 | 0.053±0.02 | 0.001±0.00 | 0.120±0.09 | 0.002±0.00 | 0.274±0.09 |
| Unidentified | 0.235 | 9.939 | 0.000±0.00 | 0.000±0.00 | 0.000±0.00 | 0.000±0.00 | 0.103±0.02 | 7.921±0.72 | 0.111±0.05 | 20.929±0.13 |
| **Charophyta** | | | | | | | | | | |

| | | | | | | | | | | |
|---|---|---|---|---|---|---|---|---|---|---|
| *Cosmarium* | 0.000 | 0.000 | 0.005±0.01 | 0.277±0.31 | 0.006±0.01 | 0.268±0.20 | 0.000±0.00 | 0.000±0.00 | 0.002±0.00 | 0.348±0.60 |
| *Staurastrum* | 0.000 | 0.000 | 0.001±0.00 | 0.083±0.07 | 0.000±0.00 | 0.006±0.01 | 0.002±0.00 | 0.155±0.18 | 0.000±0.00 | 0.034±0.06 |
| **Dinoflagellata subphylum Myzozoa** | | | | | | | | | | |
| *Ceratium* | 1.003 | 42.396 | 0.000±0.00 | 0.000±0.00 | 0.000±0.00 | 0.000±0.00 | 0.000±0.00 | 0.000±0.00 | 0.000±0.00 | 0.000±0.00 |
| *Peridinium* | 0.000 | 0.000 | 0.006±0.01 | 0.606±1.05 | 0.009±0.02 | 0.429±0.74 | 0.004±0.01 | 0.281±0.49 | 0.013±0.01 | 1.935±1.69 |

Table S2. The biomass (mg/L) and relative biomass (%) of identified taxa of phytoplankton in the invaded group in water body 1.

| **Phylum and subphylum** | **Day 1** | | **Last day** | | | | | | | |
|---|---|---|---|---|---|---|---|---|---|---|
| | | | 0 g/L NaCl | | 0.2 g/L NaCl | | 1 g/L NaCl | | 5 g/L NaCl | |
| | Biomass, mg/L | Biomass, % | Biomass, mg/L | Biomass, % | Biomass, mg/L | Biomass, % | Biomass, mg/L | Biomass, % | Biomass, mg/L | Biomass, % |
| **Cyanobacteria** | | | | | | | | | | |
| *Chroococcus* | 0.005 | 0.142 | 0.011±0.01 | 0.405±0.36 | 0.006±0.01 | 0.206±0.36 | 0.053±0.03 | 1.962±0.97 | 0.000±0.00 | 0.012±0.02 |
| *Planktolyngbya* | 0.000 | 0.000 | 0.009±0.02 | 0.361±0.58 | 0.012±0.01 | 0.403±0.52 | 0.013±0.02 | 0.552±0.78 | 0.000±0.00 | 0.000±0.00 |
| *Pseudanabaena* | 0.000 | 0.000 | 0.010±0.02 | 0.352±0.61 | 0.000±0.00 | 0.000±0.00 | 0.000±0.00 | 0.000±0.00 | 0.000±0.00 | 0.000±0.00 |
| *Romeria* | 0.000 | 0.000 | 0.002±0.00 | 0.064±0.11 | 0.001±0.00 | 0.037±0.05 | 0.001±0.00 | 0.028±0.04 | 0.000±0.00 | 0.000±0.00 |
| Unidentified | 0.000 | 0.000 | 0.019±0.03 | 0.722±1.25 | 0.000±0.00 | 0.000±0.00 | 0.000±0.00 | 0.000±0.00 | 0.000±0.00 | 0.000±0.00 |
| **Heterokontophyta subphylum Ochrophytina** | | | | | | | | | | |
| *Bitrichia* | 0.002 | 0.062 | 0.002±0.00 | 0.069±0.06 | 0.001±0.00 | 0.015±0.01 | 0.000±0.00 | 0.006±0.00 | 0.000±0.00 | 0.000±0.00 |
| *Dinobryon* | 0.016 | 0.494 | 0.017±0.03 | 0.602±1.04 | 0.000±0.00 | 0.000±0.00 | 0.000±0.00 | 0.000±0.00 | 0.000±0.00 | 0.000±0.00 |
| *Ochromonas* | 0.000 | 0.000 | 0.000±0.00 | 0.000±0.00 | 0.000±0.00 | 0.000±0.00 | 0.000±0.00 | 0.000±0.00 | 0.001±0.00 | 0.045±0.08 |
| Unidentified | 0.343 | 10.722 | 0.380±0.26 | 14.405±10.06 | 0.578±0.04 | 19.173±2.73 | 0.000±0.00 | 0.000±0.00 | 0.003±0.01 | 0.245±0.42 |
| **Heterokontophyta subphylum Bacillariophytina** | | | | | | | | | | |
| *Cyclotella* | 0.480 | 15.026 | 1.017±0.19 | 37.461±5.76 | 1.320±0.46 | 42.463±8.42 | 1.231±0.30 | 46.769±6.64 | 0.305±0.06 | 22.016±5.73 |
| *Fragilaria* | 0.000 | 0.000 | 0.002±0.00 | 0.061±0.11 | 0.001±0.00 | 0.044±0.08 | 0.000±0.00 | 0.000±0.00 | 0.000±0.00 | 0.020±0.04 |
| *Navicula* | 0.000 | 0.000 | 0.002±0.00 | 0.058±0.10 | 0.000±0.00 | 0.000±0.00 | 0.000±0.00 | 0.000±0.00 | 0.000±0.00 | 0.000±0.00 |
| *Nitzschia* | 0.001 | 0.030 | 0.082±0.02 | 3.010±0.76 | 0.045±0.02 | 1.485±0.74 | 0.021±0.00 | 0.815±0.00 | 0.027±0.02 | 1.804±1.31 |
| *Synedra* | 0.011 | 0.344 | 0.218±0.09 | 7.960±2.82 | 0.156±0.07 | 5.069±2.14 | 0.024±0.02 | 0.882±0.68 | 0.004±0.00 | 0.289±0.25 |
| **Heterokontophyta subphylum Coscinodiscophytina** | | | | | | | | | | |
| *Aulacoseira* | 0.039 | 1.226 | 0.028±0.01 | 1.026±0.50 | 0.030±0.01 | 0.981±0.47 | 0.014±0.01 | 0.512±0.38 | 0.014±0.01 | 1.094±1.02 |
| **Chlorophyta subphylum Chlorophytina** | | | | | | | | | | |
| *Chlorella* | 0.000 | 0.000 | 0.040±0.07 | 1.399±2.42 | 0.000±0.00 | 0.000±0.00 | 0.032±0.05 | 1.147±1.62 | 0.0038±0.03 | 2.591±2.30 |
| *Crucigeniella* | 0.000 | 0.000 | 0.001±0.00 | 0.023±0.04 | 0.000±0.00 | 0.000±0.00 | 0.000±0.00 | 0.000±0.00 | 0.000±0.00 | 0.029±0.05 |
| *Eutetramorus* | 0.000 | 0.000 | 0.000±0.00 | 0.008±0.01 | 0.000±0.00 | 0.000±0.00 | 0.000±0.00 | 0.000±0.00 | 0.000±0.00 | 0.000±0.00 |
| *Monoraphidium* | 0.007 | 0.228 | 0.013±0.02 | 0.451±0.50 | 0.005±0.00 | 0.154±0.06 | 0.012±0.01 | 0.491±0.28 | 0.013±0.01 | 0.889±0.39 |

| | | | | | | | | | | |
|---|---|---|---|---|---|---|---|---|---|---|
| *Nephrochlamys* | 0.104 | 3.264 | 0.031±0.03 | 1.169±1.11 | 0.049±0.03 | 1.554±0.77 | 0.039±0.03 | 1.447±0.83 | 0.022±0.01 | 1.554±0.60 |
| *Oocystis* | 0.012 | 0.371 | 0.000±0.00 | 0.000±0.00 | 0.000±0.00 | 0.000±0.00 | 0.000±0.00 | 0.000±0.00 | 0.000±0.00 | 0.019±0.03 |
| *Pediastrum* | 0.000 | 0.000 | 0.011±0.01 | 0.392±0.46 | 0.011±0.01 | 0.348±0.18 | 0.006±0.00 | 0.229±0.00 | 0.002±0.00 | 0.117±0.12 |
| *Radiococcus* | 0.000 | 0.000 | 0.001±0.00 | 0.028±0.048 | 0.000±0.00 | 0.000±0.00 | 0.000±0.00 | 0.000±0.00 | 0.000±0.00 | 0.000±0.00 |
| *Scenedesmus* | 0.066 | 2.057 | 0.185±0.08 | 6.891±3.34 | 0.079±0.03 | 2.662±1.32 | 0.044±0.01 | 1.702±0.70 | 0.104±0.03 | 7.319±1.24 |
| *Tetraedron* | 0.032 | 1.003 | 0.014±0.02 | 0.530±0.71 | 0.015±0.02 | 0.427±0.56 | 0.022±0.02 | 0.792±0.78 | 0.015±0.01 | 1.065±0.55 |
| *Tetrastrum* | 0.009 | 0.297 | 0.004±0.00 | 0.127±0.11 | 0.001±0.00 | 0.022±0.01 | 0.002±0.00 | 0.059±0.01 | 0.002±0.00 | 0.143±0.02 |
| Unidentified | 0.235 | 7.354 | 0.049±0.08 | 1.855±3.03 | 0.000±0.00 | 0.000±0.00 | 0.180±0.14 | 7.206±6.15 | 0.76±0.04 | 5.411±2.70 |
| **Charophyta** | | | | | | | | | | |
| *Closterium* | 0.000 | 0.000 | 0.001±0.00 | 0.037±0.06 | 0.004±0.01 | 0.098±0.17 | 0.006±0.01 | 0.222±0.31 | 0.008±0.01 | 0.517±0.58 |
| *Cosmarium* | 0.000 | 0.000 | 0.001±0.00 | 0.055±0.10 | 0.002±0.00 | 0.081±0.10 | 0.003±0.00 | 0.115±0.04 | 0.000±0.00 | 0.000±0.00 |
| *Staurastrum* | 0.000 | 0.000 | 0.001±0.00 | 0.049±0.02 | 0.002±0.00 | 0.077±0.03 | 0.002±0.00 | 0.095±0.00 | 0.001±0.00 | 0.046±0.08 |
| **Dinoflagellata subphylum Myzozoa** | | | | | | | | | | |
| *Ceratium* | 1.003 | 31.367 | 0.000±0.00 | 0.000±0.00 | 0.000±0.00 | 0.000±0.00 | 0.000±0.00 | 0.000±0.00 | 0.000±0.00 | 0.000±0.00 |
| *Peridinium* | 0.000 | 0.000 | 0.025±0.02 | 0.926±0.85 | 0.081±0.04 | 2.611±1.23 | 0.036±0.03 | 1.305±1.20 | 0.045±0.01 | 3.308±0.99 |
| **Invader** | | | | | | | | | | |
| *C. bergii* | 0.832 | 26.013 | 0.530±0.162 | 19.445±5.03 | 0.655±0.15 | 22.089±7.01 | 0.872±0.05 | 33.662±5.42 | 0.722±0.11 | 51.321±2.19 |

Table S3. The biomass (mg/L) and relative biomass (%) of identified taxa of phytoplankton in the control group in water body 2.

| **Phylum and subphylum** | **Day 1** | | **Last day** | | | | | | | |
|---|---|---|---|---|---|---|---|---|---|---|
| | | | 0 g/L NaCl | | 0.2 g/L NaCl | | 1 g/L NaCl | | 5 g/L NaCl | |
| | Biomass, mg/L | Biomass, % | Biomass, mg/L | Biomass, % | Biomass, mg/L | Biomass, % | Biomass, mg/L | Biomass, % | Biomass, mg/L | Biomass, % |
| **Cyanobacteria** | | | | | | | | | | |
| *Aphanocapsa* | 0.081 | 4.958 | 0.011±0.00 | 1.089±0.26 | 0.009±0.00 | 0.803±0.11 | 0.008±0.00 | 0.773±0.14 | 0.012±0.00 | 1.360±0.11 |
| *Chroococcus* | 0.011 | 0.676 | 0.010±0.00 | 0.985±0.28 | 0.008±0.00 | 0.718±0.16 | 0.008±0.00 | 0.755±0.22 | 0.013±0.01 | 1.420±0.07 |
| *Cyanodyctyon* | 0.252 | 15.500 | 0.000±0.00 | 0.026±0.01 | 0.001±0.00 | 0.054±0.04 | 0.000±0.00 | 0.000±0.00 | 0.000±0.00 | 0.000±0.00 |
| *Planktolyngbya* | 0.003 | 0.161 | 0.097±0.04 | 9.187±2.43 | 0.089±0.01 | 8.347±0.43 | 0.059±0.01 | 5.739±0.88 | 0.143±0.04 | 16.595±0.68 |
| *Romeria* | 0.000 | 0.000 | 0.000±0.00 | 0.017±0.03 | 0.001±0.00 | 0.126±0.15 | 0.000±0.00 | 0.044±0.08 | 0.000±0.00 | 0.042±0.00 |
| **Heterokontophyta subphylum Ochrophytina** | | | | | | | | | | |
| *Bitrichia* | 0.000 | 0.000 | 0.000±0.00 | 0.015±0.03 | 0.000±0.00 | 0.000±0.00 | 0.000±0.00 | 0.000±0.00 | 0.000±0.00 | 0.000±0.00 |
| *Ophiocytium* | 0.000 | 0.000 | 0.005±0.01 | 0.513±0.52 | 0.013±0.00 | 1.165±0.14 | 0.008±0.00 | 0.827±0.30 | 0.007±0.01 | 0.683±0.04 |

| | | | | | | | | | | |
|---|---|---|---|---|---|---|---|---|---|---|
| Unidentified | 0.431 | 26.494 | 0.078±0.01 | 7.834±2.07 | 0.040±0.01 | 3.704±0.17 | 0.007±0.01 | 0.671±1.16 | 0.000±0.00 | 0.000±0.00 |
| **Heterokontophyta subphylum Bacillariophytina** | | | | | | | | | | |
| *Cyclotella* | 0.247 | 15.184 | 0.067±0.03 | 6.169±2.12 | 0.077±0.01 | 7.217±0.53 | 0.203±0.07 | 20.176±7.50 | 0.056±0.04 | 5.796±0.59 |
| *Navicula* | 0.000 | 0.000 | 0.000±0.00 | 0.000±0.00 | 0.010±0.01 | 0.879±1.24 | 0.006±0.01 | 0.548±0.95 | 0.000±0.00 | 0.000±0.00 |
| *Nitzschia* | 0.000 | 0.000 | 0.057±0.05 | 5.022±3.58 | 0.069±0.04 | 6.204±2.50 | 0.041±0.02 | 4.018±1.48 | 0.003±0.00 | 0.397±0.24 |
| **Heterokontophyta subphylum Coscinodiscophytina** | | | | | | | | | | |
| *Aulacoseira* | 0.027 | 1.646 | 0.033±0.04 | 2.783±3.29 | 0.032±0.00 | 3.036±0.70 | 0.000±0.00 | 0.000±0.00 | 0.000±0.00 | 0.000±0.00 |
| **Chlorophyta subphylum Chlorophytina** | | | | | | | | | | |
| *Crucigenia* | 0.006 | 0.376 | 0.000±0.00 | 0.000±0.00 | 0.000±0.00 | 0.010±0.01 | 0.000±0.00 | 0.003±0.01 | 0.000±0.00 | 0.025±0.00 |
| *Eutetramorus* | 0.000 | 0.000 | 0.000±0.00 | 0.000±0.00 | 0.001±0.00 | 0.095±0.13 | 0.000±0.00 | 0.000±0.00 | 0.000±0.00 | 0.000±0.00 |
| *Golenkinia* | 0.000 | 0.000 | 0.001±0.00 | 0.110±0.07 | 0.001±0.00 | 0.072±0.00 | 0.001±0.00 | 0.092±0.04 | 0.000±0.00 | 0.040±0.00 |
| *Lagerheimia* | 0.000 | 0.000 | 0.001±0.00 | 0.090±0.05 | 0.001±0.00 | 0.115±0.05 | 0.001±0.00 | 0.125±0.04 | 0.002±0.00 | 0.216±0.05 |
| *Monoraphidium* | 0.010 | 0.634 | 0.078±0.01 | 7.950±2.72 | 0.065±0.01 | 6.056±0.23 | 0.055±0.02 | 5.403±1.42 | 0.045±0.01 | 4.978±0.14 |
| *Nephrochlamys* | 0.000 | 0.000 | 0.002±0.00 | 0.168±0.15 | 0.002±0.00 | 0.214±0.01 | 0.002±0.00 | 0.240±0.21 | 0.000±0.00 | 0.000±0.00 |
| *Oocystis* | 0.013 | 0.783 | 0.018±0.01 | 1.753±0.74 | 0.029±0.01 | 2.633±037 | 0.014±0.01 | 1.397±0.45 | 0.023±0.01 | 2.468±0.34 |
| *Pediastrum* | 0.002 | 0.136 | 0.151±0.06 | 14.308±3.67 | 0.108±0.04 | 9.947±1.79 | 0.125±0.07 | 12.338±7.59 | 0.056±0.07 | 7.299±1.50 |
| *Scenedesmus* | 0.207 | 12.734 | 0.091±0.01 | 8.906±1.07 | 0.116±0.01 | 10.827±0.52 | 0.154±0.01 | 15.163±0.23 | 0.238±0.08 | 25.598±1.41 |
| *Tetrachlorella* | 0.007 | 0.411 | 0.002±0.00 | 0.168±0.11 | 0.001±0.00 | 0.113±0.07 | 0.001±0.00 | 0.105±0.09 | 0.002±0.00 | 0.232±0.01 |
| *Tetraedron* | 0.148 | 9.088 | 0.174±0.04 | 16.892±1.38 | 0.196±0.00 | 18.462±2.60 | 0.111±0.01 | 10.966±1.65 | 0.177±0.10 | 18.447±2.47 |
| *Tetrastrum* | 0.000 | 0.000 | 0.005±0.00 | 0.496±0.32 | 0.003±0.00 | 0.293±0.04 | 0.004±0.00 | 0.339±0.17 | 0.007±0.00 | 0.793±0.06 |
| Unidentified | 0.181 | 11.091 | 0.141±0.03 | 14.302±4.90 | 0.172±0.01 | 16.120±1.96 | 0.177±0.05 | 17.279±3.76 | 0.116±0.04 | 12.506±1.11 |
| **Charophyta** | | | | | | | | | | |
| *Cosmarium* | 0.000 | 0.000 | 0.012±0.01 | 0.496±0.32 | 0.021±0.01 | 1.920±0.49 | 0.016±0.01 | 1.602±0.77 | 0.011±0.01 | 1.103±0.18 |
| *Staurastrum* | 0.002 | 0.129 | 0.141±0.03 | 14.302±4.90 | 0.009±0.00 | 0.869±0.13 | 0.011±0.01 | 1.106±0.58 | 0.000±0.00 | 0.000±0.00 |
| **Dinoflagellata subphylum Myzozoa** | | | | | | | | | | |
| *Peridinium* | 0.000 | 0.000 | 0.000±0.00 | 0.000±0.00 | 0.000±0.00 | 0.000±0.00 | 0.003±0.01 | 0.294±0.51 | 0.000±0.00 | 0.000±0.00 |

Table S4. The biomass (mg/L) and relative biomass (%) of identified taxa of phytoplankton in the invaded group in water body 2.

| Phylum and subphylum | Day 1 | | Last day |
|---|---|---|---|

| | Biomass, mg/L | Biomass, % | 0 g/L NaCl Biomass, mg/L | 0 g/L NaCl Biomass, % | 0.2 g/L NaCl Biomass, mg/L | 0.2 g/L NaCl Biomass, % | 1 g/L NaCl Biomass, mg/L | 1 g/L NaCl Biomass, % | 5 g/L NaCl Biomass, mg/L | 5 g/L NaCl Biomass, % |
|---|---|---|---|---|---|---|---|---|---|---|
| **Cyanobacteria** | | | | | | | | | | |
| *Aphanocapsa* | 0.081 | 3.720 | 0.012±0.01 | 0.760±0.23 | 0.011±0.00 | 0.512±0.09 | 0.007±0.00 | 0.237±0.05 | 0.009±0.00 | 0.227±0.11 |
| *Chroococcus* | 0.011 | 0.508 | 0.008±0.00 | 0.555±0.24 | 0.022±0.01 | 0.680±0.51 | 0.022±0.02 | 0.649±0.37 | 0.014±0.00 | 0.369±0.07 |
| *Cyanodyctyon* | 0.252 | 11.630 | 0.000±0.00 | 0.014±0.02 | 0.000±0.00 | 0.000±0.00 | 0.000±0.00 | 0.001±0.00 | 0.000±0.00 | 0.000±0.00 |
| *Planktolyngbya* | 0.003 | 0.120 | 0.083±0.04 | 5.499±2.15 | 0.020±0.01 | 0.896±0.06 | 0.076±0.03 | 2.791±1.73 | 0.160±0.04 | 4.164±0.68 |
| *Romeria* | 0.000 | 0.000 | 0.000±0.00 | 0.008±0.01 | 0.000±0.00 | 0.000±0.00 | 0.000±0.00 | 0.002±0.00 | 0.000±0.00 | 0.000±0.00 |
| **Heterokontophyta subphylum Ochrophytina** | | | | | | | | | | |
| *Bitrichia* | 0.000 | 0.000 | 0.000±0.00 | 0.025±0.02 | 0.000±0.00 | 0.013±0.00 | 0.000±0.00 | 0.000±0.00 | 0.000±0.00 | 0.000±0.00 |
| *Ophiocytium* | 0.000 | 0.000 | 0.007±0.01 | 0.519±0.65 | 0.122±0.16 | 6.911±9.43 | 0.189±0.32 | 4.464±7.30 | 0.002±0.00 | 0.049±0.04 |
| Unidentified | 0.431 | 19.880 | 0.059±0.10 | 3.291±5.70 | 0.046±0.02 | 2.290±1.70 | 0.035±0.02 | 1.233±0.70 | 0.000±0.00 | 0.000±0.00 |
| **Heterokontophyta subphylum Bacillariophytina** | | | | | | | | | | |
| *Cyclotella* | 0.247 | 11.394 | 0.104±0.02 | 6.920±1.33 | 0.118±0.05 | 5.838±4.28 | 0.120±0.02 | 4.212±1.80 | 0.098±0.02 | 2.568±0.59 |
| *Nitzschia* | 0.000 | 0.000 | 0.030±0.02 | 1.954±1.06 | 0.081±0.04 | 4.103±3.32 | 0.031±0.02 | 0.914±0.54 | 0.008±0.01 | 0.214±0.24 |
| **Heterokontophyta subphylum Coscinodiscophytina** | | | | | | | | | | |
| *Aulacoseira* | 0.027 | 1.235 | 0.015±0.00 | 0.954±0.10 | 0.059±0.07 | 3.279±4.34 | 0.000±0.00 | 0.000±0.00 | 0.000±0.00 | 0.000±0.00 |
| **Chlorophyta subphylum Chlorophytina** | | | | | | | | | | |
| *Crucigenia* | 0.006 | 0.282 | 0.000±0.00 | 0.07±0.01 | 0.000±0.00 | 0.013±0.02 | 0.000±0.00 | 0.000±0.00 | 0.000±0.00 | 0.000±0.00 |
| *Eutetramorus* | 0.000 | 0.000 | 0.000±0.00 | 0.000±0.00 | 0.000±0.00 | 0.000±0.00 | 0.001±0.00 | 0.030±0.05 | 0.000±0.00 | 0.000±0.00 |
| *Golenkinia* | 0.000 | 0.000 | 0.001±0.00 | 0.087±0.04 | 0.002±0.00 | 0.084±0.00 | 0.001±0.00 | 0.029±0.03 | 0.000±0.00 | 0.013±0.00 |
| *Lagerheimia* | 0.000 | 0.000 | 0.001±0.00 | 0.056±0.07 | 0.000±0.00 | 0.014±0.02 | 0.000±0.00 | 0.016±0.00 | 0.003±0.00 | 0.076±0.05 |
| *Monoraphidium* | 0.010 | 0.476 | 0.051±0.02 | 3.454±1.24 | 0.274±0.32 | 10.166±10.71 | 0.387±0.57 | 9.649±12.58 | 0.054±0.00 | 1.432±0.14 |
| *Nephrochlamys* | 0.000 | 0.000 | 0.004±0.00 | 0.293±0.29 | 0.000±0.00 | 0.000±0.00 | 0.001±0.00 | 0.039±0.02 | 0.000±0.00 | 0.000±0.00 |
| *Oocystis* | 0.013 | 0.587 | 0.018±0.01 | 1.147±0.36 | 0.031±0.02 | 1.288±0.27 | 0.015±0.00 | 0.497±0.14 | 0.020±0.01 | 0.523±0.34 |
| *Pediastrum* | 0.002 | 0.102 | 0.103±0.06 | 6.495±3.52 | 0.056±0.00 | 2.526±0.64 | 0.214±0.03 | 7.170±1.89 | 0.103±0.05 | 2.781±1.50 |
| *Scenedesmus* | 0.207 | 9.555 | 0.101±0.01 | 6.771±1.55 | 0.157±0.06 | 6.813±0.30 | 0.175±0.05 | 5.626±0.35 | 0.269±0.05 | 7.087±1.41 |
| *Tetrachlorella* | 0.007 | 0.308 | 0.001±0.00 | 0.043±0.01 | 0.001±0.00 | 0.062±0.01 | 0.001±0.00 | 0.056±0.06 | 0.001±0.00 | 0.019±0.01 |

| | | | | | | | | | | |
|---|---|---|---|---|---|---|---|---|---|---|
| *Tetraedron* | 0.148 | 6.819 | 0.146±0.07 | 9.242±2.55 | 0.152±0.05 | 6.643±0.23 | 0.171±0.04 | 5.594±0.74 | 0.269±0.10 | 7.039±2.47 |
| *Tetrastrum* | 0.000 | 0.000 | 0.003±0.00 | 0.215±0.08 | 0.004±0.00 | 0.195±0.03 | 0.005±0.00 | 0.156±0.08 | 0.006±0.00 | 0.144±0.06 |
| Unidentified | 0.181 | 8.322 | 0.132±0.02 | 8.869±2.23 | 0.125±0.08 | 5.138±1.93 | 0.142±0.02 | 4.407±1.14 | 0.098±0.03 | 2.624±1.11 |
| **Charophyta** | | | | | | | | | | |
| *Cosmarium* | 0.000 | 0.000 | 0.008±0.00 | 0.535±0.08 | 0.012±0.00 | 0.508±0.00 | 0.035±0.03 | 1.279±1.23 | 0.018±0.01 | 0.479±0.18 |
| *Staurastrum* | 0.002 | 0.096 | 0.002±0.00 | 0.103±0.01 | 0.000±0.00 | 0.000±0.00 | 0.001±0.00 | 0.026±0.02 | 0.000±0.00 | 0.000±0.00 |
| **Dinoflagellata subphylum Myzozoa** | | | | | | | | | | |
| *Peridinium* | 0.000 | 0.000 | 0.038±0.07 | 2.114±3.66 | 0.008±0.01 | 0.275±0.39 | 0.008±0.01 | 0.310±0.54 | 0.000±0.00 | 0.000±0.00 |
| **Invader** | | | | | | | | | | |
| *S. aphanizomenoides* | 0.542 | 24.964 | 0.603±0.18 | 38.570±13.68 | 0.992±0.56 | 41.473±10.36 | 1.494±0.11 | 50.186±11.17 | 2.676±0.28 | 70.190±3.83 |
